# Supplementary material for: Exploring barriers for trachomatous trichiasis surgery implementation in gamo zone, Southern Ethiopia
Source: PLoS Negl Trop Dis. 2021 Sep 15;15(9):e0009780. doi: 10.1371/journal.pntd.0009780 (PMC8476041; doi:10.1371/journal.pntd.0009780)
Supplement: Attachment — Submitted filename: Response to_review comments22.docx [file pntd.0009780.s001.docx]

**RESPONSE TO EDITORIAL AND REVIEWERS COMMENTS**

| Date: | Jul 17 2021 |
| --- | --- |
| To: | "PLOS Neglected Tropical Diseases" plosntds@plos.org |
| From: | "CHUCHU CHURKO" [churkochuchu2005@gmail.com](mailto:churkochuchu2005@gmail.com) |
| Subject: | Response to reviewers comments (PNTD-D-21-00777) |

**Dear Editor and Reviewers**

Thank you very much for your fruitful and constructive comments on our manuscript "EXPLORING BARRIERS FOR TRICHIASIS SURGERY IMPLEMENTATION IN GAMO ZONE, SOUTHERN ETHIOPIA, 2019". We have provided point by point response for reviewers’ comments and described changes that we have made in the revised manuscript. We have also submitted the two versions of the revised manuscript; one with track changes and the other the cleaned version.

Sincerely,

Chuchu Churko

Associate researcher at Arba Minch University

***********************

**AUTHORS’ RESPONSE TO REVIEWERS COMMENTS**

**Methods**

-Are the objectives of the study clearly articulated with a clear testable hypothesis stated?

-Is the study design appropriate to address the stated objectives?

-Is the population clearly described and appropriate for the hypothesis being tested?

-Is the sample size sufficient to ensure adequate power to address the hypothesis being tested?

-Were correct statistical analysis used to support conclusions?

-Are there concerns about ethical or regulatory requirements being met?

Reviewer #1: The objectives of the study are clearly stated. The study population is clearly described. The sample size is also acceptable for such a qualitative study. The FGDs and in-depth interviews were fairly transcribed and analyzed but lacks clarity due to weak translation into English language. Ethical requirements were strictly observed.

Reviewer #2: --No hypothesis but this is a qualitative study

--Study design appropriate but unclear that best practices were followed

--population not clearly described, how people were chosen is not clear

--no discussion about sample size calculations so unclear if the sample size is sufficient

--------------------

**Authors’ response:** we appreciated reviewers for their constructive comments on the method section. All the authors tried to revise manuscript for English language editing. **Reviewer #2** raised his concern on study design ‘Study design appropriate but unclear that best practices were followed’. We are not sure whether the following response satisfies our reviewer concern. Key informants and FGDs were used to collect important information about barriers that affect the implementation of trichiasis surgery. We developed semi-structured KIIs and FGDs interview topics and pretested for any modification and adjustments were made to gather the data. Translation and transcriptions were made by experts like University instructors who were fluent in local and English language. The analysis of the data was made by manually through thematic content analysis. These are the best practices that should be followed for qualitative study. **Reviewer 2** also asked to clarify how the study population were chosen. The authors indicated this point in the revised manuscript that the study population were selected by using purposive sampling technique. For FGDs: Trichiasis patients were screened from the community IECWs and invited to attend the discussion after specifying the meeting time and site. Previously operated patients were identified from TT operated patient card in the health facility. Then, the IECWs went to their communities and appointed them for discussion after informing the objective of the study. The other group was health extension group. They were called to come each health office for discussion by the NTD focal person of the district from kebeles where the disease is more prevalent.

**Reviewer 2** also asked to clarify about sample size calculation. Since this is qualitative study the authors did not use sample size calculation, rather the authors predetermined the number of FGDs and KIIs and was planned to conduct 9 FGDs and 12 KIIs. Based on this regard, 9 FGDs and 12 in-depth interviews were conducted.

**Results**

-Does the analysis presented match the analysis plan?

-Are the results clearly and completely presented?

-Are the figures (Tables, Images) of sufficient quality for clarity

Reviewer #1: Results were not presented in accordance with the analysis plan. Results of the 3 different FGDs as categorized by their affiliations (operated TT cases, unoperated TT cases and IECWs) should have been presented separately so that the barriers for TT surgery as perceived by these 3 different groups could be clearly understood by readers of the article. Similarly, the key informant interviews should have been presented separately so that the view points expressed by service providers could be clearly understood. The authors have presented the results by thematic areas which makes it difficult for readers to clearly and succinctly identify the real barriers to TT surgery. Recurrent trichiasis appears to be a major problem in the study area but the authors didn't adequately address this critical issue. Overall, the data presented are too shallow and anecdotal. I don't think that the authors have presented strong evidence to make meaningful inferences.

Reviewer #2: --No analysis plan was given. But the pre-specified analysis plan would be a helpful addition

--Results are clear but it is not certain how representative the results are given the lack of clarity on the analysis methods

--------------------
**Authors’ response:** the authors accepted the reviewers’ comments. The result section is amended and written separately based on the 3 different FGDs. The KIIs is also written separately to make it easy for readers and understand the barriers to TT surgery. We have included some published literatures in the discussion section that support our findings on recurrence of trichiasis. The analysis plan is described in data processing and analysis section of the revised manuscript.

**Conclusions**

-Are the conclusions supported by the data presented?

-Are the limitations of analysis clearly described?

-Do the authors discuss how these data can be helpful to advance our understanding of the topic under study?

-Is public health relevance addressed?


Reviewer #1: The conclusions are supported by the data presented and the limitations of the study are also described. However, there is a serious problem with the English language.

Reviewer #2: --limitations can be expanded depending on the details of the analysis (see comments below)

**Authors’ response:** The authors have completely revised the manuscript for English language editing.
--------------------

**Editorial and Data Presentation Modifications?**


Use this section for editorial suggestions as well as relatively minor modifications of existing data that would enhance clarity. If the only modifications needed are minor and/or editorial, you may wish to recommend “Minor Revision” or “Accept”.

Reviewer #1: (No Response)

Reviewer #2: (No Response)

--------------------

**Summary and General Comments**


Use this section to provide overall comments, discuss strengths/weaknesses of the study, novelty, significance, general execution and scholarship. You may also include additional comments for the author, including concerns about dual publication, research ethics, or publication ethics. If requesting major revision, please articulate the new experiments that are needed.

Reviewer #1: The study addresses an important problem for trachoma elimination program. The method used is acceptable. However, the data and evidence presented is so weak and superficial. The data synthesis and analysis also lacks clarity and depth. The manuscript needs complete revision.

Reviewer #2: This is a qualitative research study assessing barriers to trachomatous trichiasis surgery. My main comment is that the authors have not demonstrated scientific rigor with their methods (please see detailed comments below). They provide some quotes from several people but it is unclear how they decided that these were the consensus views of the community. Other comments follow:

**Authors’ response:** the authors tried to make complete revision of the manuscript. Please see the revised version of the manuscript. Thanks!!!

Abstract, background: It might be better to specify “trachomatous trichiasis” as the leading infectious cause
**Authors’ response**: We have changed ‘’Trichiasis is the leading cause of blindness’’ to ‘’ Trachomatous trichiasis is the leading infectious cause of blindness’’

Abstract, findings: non-Ethiopians will not know what a “kebeles” is; please substitute “communities”
**Authors’ response:** we have amended the word ‘’kebeles’’ to ‘’communities’’

Abstract, conclusion: “the main barriers for trichiasis surgery implementation were from service providers and patients’ side.” ◊ As opposed to what?
**Authors’ response:** in the abstract conclusion section, the typological errors have been amended. Please see the revised version of the manuscript.

Introduction: trachoma is not the leading cause of blindness. Cataract is. You could say “leading infectious cause”?
**Authors’ response:** This sentence is revised as follows ‘’ Trachoma is the leading infectious cause of blindness worldwide.’’

How was the sample size determined? Were the numbers pre-specified? Or continued until saturation was reached?
**Authors’ response:** Since this is qualitative study the authors did not use sample size calculation, rather the authors predetermined the number of FGDs and KIIs. Based on this regard, 9 FGDs and 12 in-depth interviews were expected to be conducted. We have included sample size calculation section in the revised manuscript.

How exactly were the participants of the FGDs selected? It says trichiasis patients. From where were they recruited? From a health clinic? From a campaign?
**Authors’ response:** the authors revised the selection of study participants and sampling procedure section to make it clearer and we hope the reviewer concern is resolved here.

Methods: The methods for analysis are not detailed. Specifically: (A) usually qualitative data is analyzed according to some underlying theoretical model. Did the authors consider any model when analyzing? If not, please state that the analysis was done without considering a specific framework. (B) was coding of transcripts done to classify responses into specific domains? If so, who did the coding? How many people? How did you assure that different graders reached similar conclusions in their coding (eg. Kappa). (C) what software was used? Any software specific to qualitative research (eg atlas.ti or something similar)
It would be good to more clearly state who did the key informant interviews (for example, who are “district key informants”?). Many people outside Ethiopia will not know what a health extension worker is, so please give a brief description of who they are and why we should care about their opinions.
**Authors’ response:-** the analysis of the data was done systematically by the theoretical content analysis approach. All Codes or categories of transcript were done by corresponding/first author. We did not use any software in this study. Both FGDs and key informant interviews were done by the trained health professionals who have previous experience on qualitative data collection. This is stated in the manuscript quality control section. As we have described in the manuscript, district key informants were health office heads and NTDs focal persons. The authors briefly described about health extension workers. Please the revised version of the manuscript.

Most readers will not know what a woreda is, please define, maybe just say “(i.e., district)”
**Authors’ response:** the word ‘woreda’ is replaced by ‘district’ throughout the revised manuscript.

Recommendation: what exactly is the recommendation? Are there any barriers that you would prioritize based on your research? Things that would be easy/cheap to do first, then followed by more complicated things?
 **Authors’ response:** we have made changes on recommendation section by adding the following: ‘’closely supervising the integrated eye care workers would be the first task for district health offices to increase the uptake and improve the quality of service. In-service training should be given to maintain the existing system and increase the number of IECWs. Logistics and supplies should be made available and adequate to address all affected people in the community.’’

--------------------
**Do you want your identity to be public for this peer review?** For information about this choice, including consent withdrawal, please see our [Privacy Policy](https://www.plos.org/privacy-policy).

Reviewer #1: No

Reviewer #2: No
