# Supplementary material for: Exploring barriers for trachomatous trichiasis surgery implementation in gamo zone, Southern Ethiopia
Source: PLoS Negl Trop Dis. 2021 Sep 15;15(9):e0009780. doi: 10.1371/journal.pntd.0009780 (PMC8476041; doi:10.1371/journal.pntd.0009780)
Supplement: Attachment — Submitted filename: AUTHORS RESPONSE2_review comments.docx [file pntd.0009780.s002.docx]

**Authors’ response to reviewer comments.**

| Date: | Aug 05 2021 09:39AM |
| --- | --- |
| To: | "PLOS Neglected Tropical Diseases" plosntds@plos.org |
| From: | "CHUCHU CHURKO" churkochuchu2005@gmail.com |
| Subject: | Authors’ response to reviewers comments on our submission to PLOS Neglected Tropical Diseases (PNTD-D-21-00777R1) |

Dear Reviewers and Editor,

Thank you very much for your constructive comments on our manuscript "EXPLORING BARRIERS FOR TRICHIASIS SURGERY IMPLEMENTATION IN GAMO ZONE, SOUTHERN ETHIOPIA, 2019". We have provided point by point response for reviewers’ comments and described changes that we have made in the revised manuscript. We have also submitted the two versions of the revised manuscript; one with track changes and the other the cleaned version.

**Methods**

-Are the objectives of the study clearly articulated with a clear testable hypothesis stated?

-Is the study design appropriate to address the stated objectives?

-Is the population clearly described and appropriate for the hypothesis being tested?

-Is the sample size sufficient to ensure adequate power to address the hypothesis being tested?

-Were correct statistical analysis used to support conclusions?

-Are there concerns about ethical or regulatory requirements being met?


Reviewer #1: I don't think the study design is appropriate to address the stated objectives. The study population has not been clearly described. The authors simply gathered some TT backlog estimates for some selected districts but didn't even present how many of those were operated. Although it is a qualitative study, the sample size of the FGDs, KIIs, etc. should fairly represent the study population. For example, there were only 21 operated TT cases in the FGDs. What was the total number of TT cases operated in the three districts? No data is given. It's not also clear if they have referred to the TT case registers in the health facilities. They were relying on the verbal confirmations provided by the NGO partner (Orbis) and some of the district health office staff.

Reviewer #2: Adequate

**Authors’ response:** as we have described in the manuscript the objective of this study was to explore barriers for implementation of trichiasis surgery. Therefore, we used qualitative study design to address our objective. I think this design is scientifically appropriate to achieve the stated objective. In the method section the authors presented who were the study subjects. We used key informants like district and health centre heads, neglected tropical diseases (NTD) focal persons and integrated eye care workers (IECWs) as key informant. For FGD, we used previously operated individuals, trachomatous trichiasis patients and health extension workers. As we know scientific recommendation for sample size calculation in qualitative study is based on idea saturation. In a research, 6 up to 12 FGDs and 10-25 KIIs are recommended to reach idea saturation. So, we conducted 9 FGDs each with 6-9 participants participated and 12 KIIs. Please see table 1 for further clarification. Obtaining number of individuals operated for trichiasis in each district was not our objective. The issue was the high number of backlog in the three districts. That was why the authors started to study the barriers. We hope our reviewer satisfy with these responses.

--------------------

**Results**
-Does the analysis presented match the analysis plan?

-Are the results clearly and completely presented?

-Are the figures (Tables, Images) of sufficient quality for clarity?

Reviewer #1: The results and the analyses presented lack clarity and completeness. The descriptions/explanations provided are weak and not analytic. They are quoting some references in the discussion section that are not relevant to the findings of the study (things like the SDGs). The quotes (translations) from the FGD discussants are not written with good English. Instead of describing the identified barriers to TT surgery, the authors were rather dwelling more on discussing other broader components of the SAFE strategy and government policies for trachoma control.

Reviewer #2: Adequate

**Authors’ response:** all authors appreciated the reviewers’ comments and suggestion. We have presented how our findings were analysed in the method section. The descriptions might be weakly provided. However, we have tried our best to make our results clear to readers. Regarding SGDs quoting, every program should be focused to achieve SGDs goals. Trachomatous trichiasis surgery is one of NTDs program aimed to eliminate blinding trachoma. This is derived from SGDs goal 3. That is why we were quoted SGDs in the discussion. We are confused on the comment ‘‘Instead of describing the identified barriers to TT surgery, the authors were rather dwelling more on discussing other broader components of the SAFE strategy and government policies for trachoma control’’ raised by reviewer 1. Out of 10 paragraphs in discussion section, none have discussing on the other broader component of SAFE strategy. Rather, the authors discussed the pertinent findings like barriers to TT surgery.

Thank you!!!

--------------------

**Conclusions**

-Are the conclusions supported by the data presented?

-Are the limitations of analysis clearly described?

-Do the authors discuss how these data can be helpful to advance our understanding of the topic under study?

-Is public health relevance addressed?

Reviewer #1: The conclusions are, to some extent, supported by the data presented. Some of the limitations of the study are stated.

Reviewer #2: Adequate

**Authors’ response:** Thank you both reviewers for the interesting comments you have made on our manuscript.

--------------------

**Editorial and Data Presentation Modifications?**

Use this section for editorial suggestions as well as relatively minor modifications of existing data that would enhance clarity. If the only modifications needed are minor and/or editorial, you may wish to recommend “Minor Revision” or “Accept”.

Reviewer #1: The paper needs thorough revision.

Reviewer #2: (No Response)

**Authors’ response:** Thank you again for your reviews. The authors thoroughly revised the manuscript. Please see the revised version of the manuscript.

--------------------

**Summary and General Comments**

Use this section to provide overall comments, discuss strengths/weaknesses of the study, novelty, significance, general execution and scholarship. You may also include additional comments for the author, including concerns about dual publication, research ethics, or publication ethics. If requesting major revision, please articulate the new experiments that are needed.
Reviewer #1: The paper needs major revision.

Reviewer #2: The authors have addressed my comments.

**Authors’ response:** revisions have been made on the manuscript.

--------------------
PLOS authors have the option to publish the peer review history of their article ([what does this mean?](https://journals.plos.org/plosntds/s/editorial-and-peer-review-process#loc-peer-review-history)). If published, this will include your full peer review and any attached files.
If you choose “no”, your identity will remain anonymous but your review may still be made public.
**Do you want your identity to be public for this peer review?** For information about this choice, including consent withdrawal, please see our [Privacy Policy](https://www.plos.org/privacy-policy).

Reviewer #1: No

Reviewer #2: No

--------------------------

**Figure Files:**

While revising your submission, please upload your figure files to the Preflight Analysis and Conversion Engine (PACE) digital diagnostic tool, https://pacev2.apexcovantage.com. PACE helps ensure that figures meet PLOS requirements. To use PACE, you must first register as a user. Then, login and navigate to the UPLOAD tab, where you will find detailed instructions on how to use the tool. If you encounter any issues or have any questions when using PACE, please email us at [figures@plos.org](mailto:figures@plos.org).

**Authors’ response:** N/A. we have no figure files.

--------------------------
**Data Requirements**:

Please note that, as a condition of publication, PLOS' data policy requires that you make available all data used to draw the conclusions outlined in your manuscript. Data must be deposited in an appropriate repository, included within the body of the manuscript, or uploaded as supporting information. This includes all numerical values that were used to generate graphs, histograms etc.. For an example see here:

http://www.plosbiology.org/article/info%3Adoi%2F10.1371%2Fjournal.pbio.1001908#s5.
**Authors’ response:** all relevant data are within the body of the manuscript.

------------------------
**Reproducibility:**
To enhance the reproducibility of your results, we recommend that you deposit your laboratory protocols in protocols.io, where a protocol can be assigned its own identifier (DOI) such that it can be cited independently in the future. Additionally, PLOS ONE offers an option to publish peer-reviewed clinical study protocols. Read more information on sharing protocols at

<https://plos.org/protocols?utm_medium=editorialemail&utm_source=authorletters&utm_campaign=protocols>
**Authors’ response**: N/A

----------------------------
**References**
Please review your reference list to ensure that it is complete and correct. If you have cited papers that have been retracted, please include the rationale for doing so in the manuscript text, or remove these references and replace them with relevant current references. Any changes to the reference list should be mentioned in the rebuttal letter that accompanies your revised manuscript. If you need to cite a retracted article, indicate the article's retracted status in the References list and also include a citation and full reference for the retraction notice.
**Authors’ response:** the authors checked all references for its completeness.

____________________
